# Supplementary material for: On the origins of conductive pulse sensing inside a nanopore
Source: Nat Commun. 2022 May 13;13:2186. doi: 10.1038/s41467-022-29758-8 (PMC9106702; doi:10.1038/s41467-022-29758-8)
Supplement: Supplementary file 1 — Supplementary Information for “On the Origins of Conductive Pulse Sensing Inside a Nanopore” [file 41467_2022_29758_MOESM1_ESM.pdf]

Supplementary Information for "On the Origins of Conductive Pulse Sensing Inside a Nanopore"

Lauren S. Lastra[1], Y. M. Nuwan D. Y. Bandara[1], Michelle Nguyen[2], Nasim Farajpour[1], Kevin J. Freedman[1]*

[1]Department of Bioengineering, University of California, Riverside, 900 University Ave., Riverside, CA 92521, USA

[2]Department of Biology, University of California, Riverside, 900 University Ave., Riverside, CA 92521, USA

*Correspondence to: Kevin J. Freedman, Department of Bioengineering, University of California, Riverside, 900 University Ave., Riverside, CA 92521, USA. E-mail: kfreedman@engr.ucr.edu

Contents

Section 1: Translocation of PEG 2
Section 2: Translocation of Transferrin 4
Section 3: TEM Characterization of Nanopipettes 4
Section 4: Signal to Noise (SNR) ratio and Pore Size Event Rate Calculation 5
Section 5: Finite Element Methods 7
Section 6: Event Rate Calculation 10
Section 7: Event Extraction and Classification of Conformations 11
Section 8: Electroosmotic versus Electrophoretic Transport 12
Section 9: Streaming Current 15
Section 10: Translocation Characteristics in LiCl 16
Section 11: Measurements in CsCl 18
Section 12: Time-Dependent Electric field and Flux Modeling 18
References 19

Section 1: Translocation of PEG

Although the PEG polymer is neutral, when added to KCl it functions like a polycationic polymer due to cation adsorption. This has been previously observed by *Reiner et. al.* in their work with α-hemolysin pores[1]. PEG 20,000 was diluted to 15% (w/w) in 10 mM KCl and a negative voltage bias was applied wherein PEG events were independently observed with six different pores. Corresponding current traces are shown in Supplementary Figure 1. Interestingly, conductive events (CEs) were most notable at extremely small pore sizes (e.g., 0.43 nS); a pore size regime that we could not observe DNA events. Because PEG is positively charged, we were able to observe events at small pore sizes through EPF unlike DNA (Supplementary Figure 2). Since EOF flow rate decreases with smaller pore sizes and EPF increases, we believe DNA could not energetically overcome the barrier at the pore entrance for translocations to occur. The results indicated that smaller pore sizes resulted in CEs whereas larger pore translocations yielded

resistive events (REs). Consequently, as shown in the main text, when EOF becomes the dominant translocation mechanism, smaller pores yield REs whereas larger pores yield CEs. However, this observation cannot be explained through the theory presented by *Zhang et. al.* and requires further exploration as demonstrated in the manuscript[2].

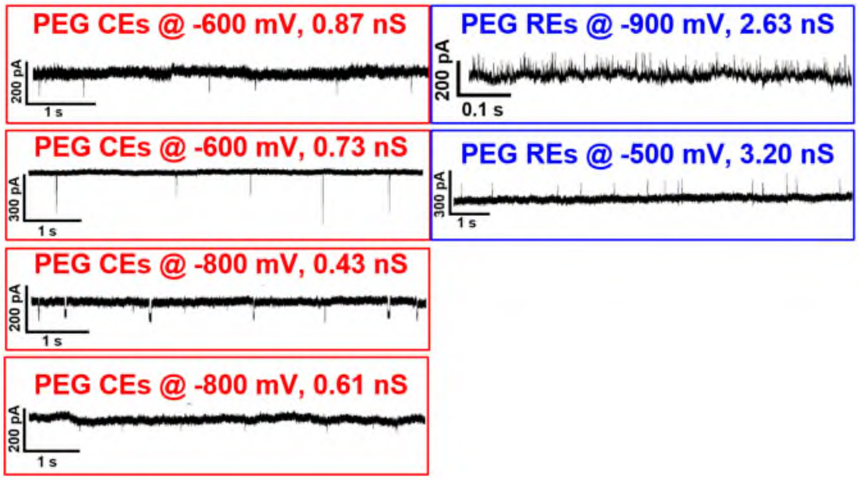

Supplementary Figure 1: Current traces of PEG translocating in 10 mM KCl in six different pores. On the left (red), are the CEs seen with smaller pores and on the right (blue) are the REs from PEG translocating through larger pores.

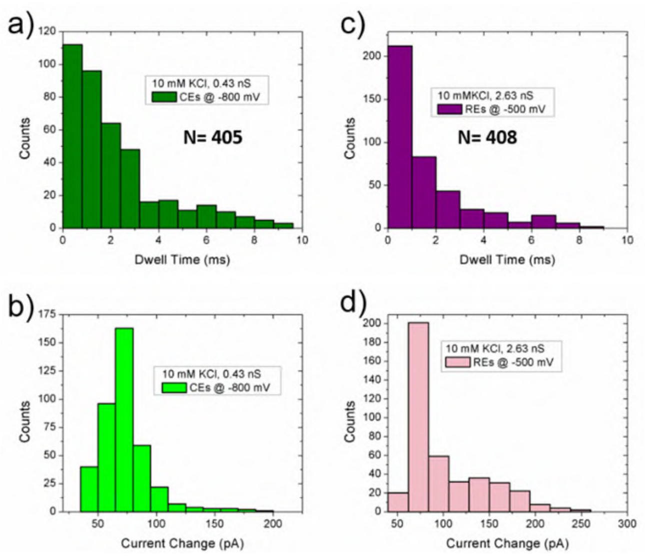

Supplementary Figure 2: Event information about PEG 20,000 in 10 mM KCl. Histograms of (**a**) dwell time and (**b**) current change for CEs with 0.43 nS pore at -800 mV. Histograms of (**c**) dwell time and (**d**) current change of REs with 2.63 nS pore at -500 mV.

Section 2: Translocation of Transferrin

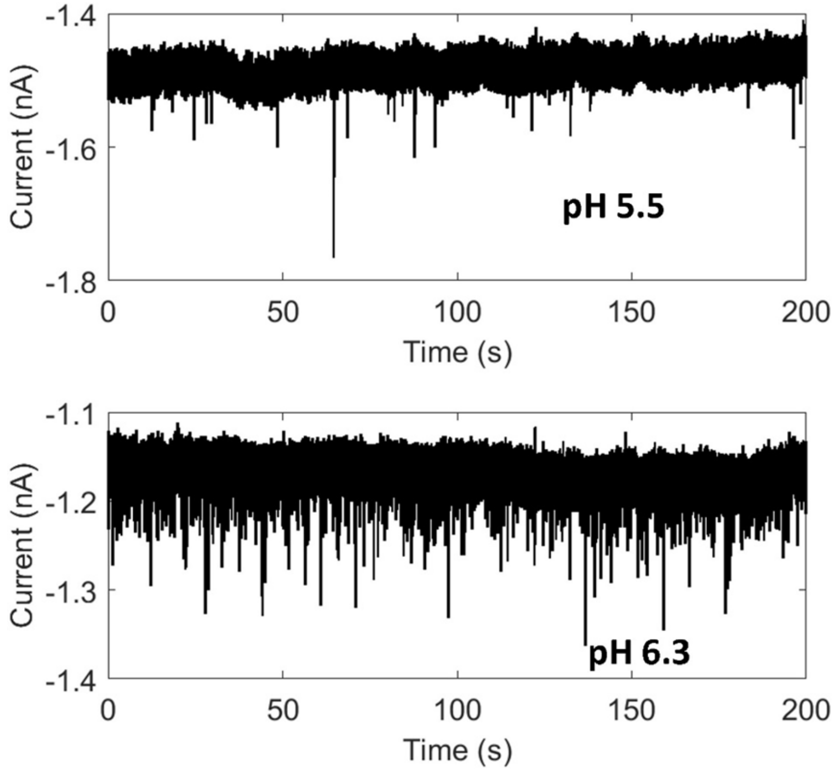

Supplementary Figure 3: Transferrin translocations through a ~30 nm diameter pore under low ionic strength conditions (10 mM KCl buffered with 1 mM PBS) at two different pH values. Transferrin concentration was 350 nM and events were recorded at -400 mV. Under both pH values (one at isoelectric point and one above), conductive events were observed.

Section 3: TEM Characterization of Nanopipettes

For TEM imaging and all experiments, nanopores were fabricated through using a laser-assisted pulling machine. To secure the nanopore tip onto the TEM grid, epoxy glue was used. Epoxy was first added into a plastic holder and the nanopore was secured underneath an optical

microscope with a microscale actuator. The TEM grid was then held by tweezers and epoxy was applied onto one side of the grid using a plastic stirring rod.

After successful application of epoxy to TEM grid, the grid (glue facing upwards) was then placed underneath optical microscope as well. With the naked eye, the nanopore was moved as close as possible to the epoxy. An optical microscope and micromanipulator were then used to ensure the nanopore tip was just inside the grid, without the pore aperture touching the epoxy. One last layer of epoxy to secure the nanopore in place was applied once nanopore was in the correct position. This setup was left to dry for 15 minutes.

Once 15 minutes has expired, the nanopore was gently broken away from the TEM grid, leaving just the pore inside the grid. Finally, a gold coating was applied to the pore and the grid for preparation of TEM imaging. Specifically, Tecnai12 (ThermoFisher Scientific) was used for imaging, with a lanthium hexaboride electron source operated at 120 kV.

Section 4: Signal to Noise (SNR) Ratio and Pore Size

For all SNR calculations, we omitted all configurations except for linear DNA translocations. DNA has the ability to translocate linearly, folded[3,4], or in knots[5]. To ensure DNA configuration had no effect on SNR, we utilized only linearly translocating DNA to our calculation. We witness an increase in SNR starting at 2.0 nS and saturating around 3.0 nS (Supplementary Figure 3). To determine whether the current enhancement or the noise of the signal is the major contributor to the increase in SNR, we acquired the median current change of all events and the root mean square (RMS) noise of a data segment lacking events. We witness that the RMS noise maintaining values of 15 ± 7 pA, whereas the current change increases from 30 to 140 pA as pore size increases. For the left side of the graph, we witness a sharp increase in the SNR as the pore size decreases. This can be explained by a decrease in the noise associated with

smaller pores. As seen in Supplementary Figure 4, the RMS noise is extremely low (< 10 pA) whereas the median current change is approximately 100 pA. Therefore, the higher SNR values for smaller pores stem from lower noise. On the right side of the graph, we speculate that the rise in SNR (and current enhancement) is a result of greater EOF pumping as a function of pore size (Supplementary Figure 5).

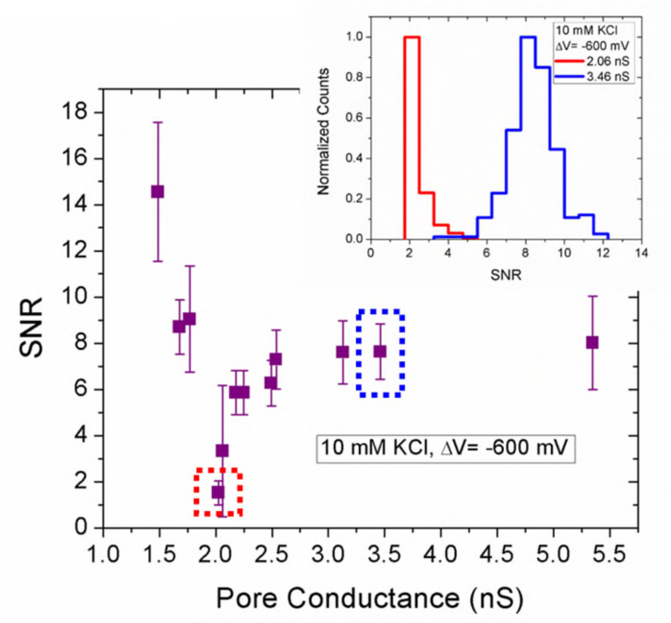

Supplementary Figure 4: **SNR with Pore Conductance.** SNR calculations for various pore sizes (inset: normalized histogram of data sets from red and blue dotted squares). Each symbol and error bar represent the mean and standard deviation of SNR for one pore.

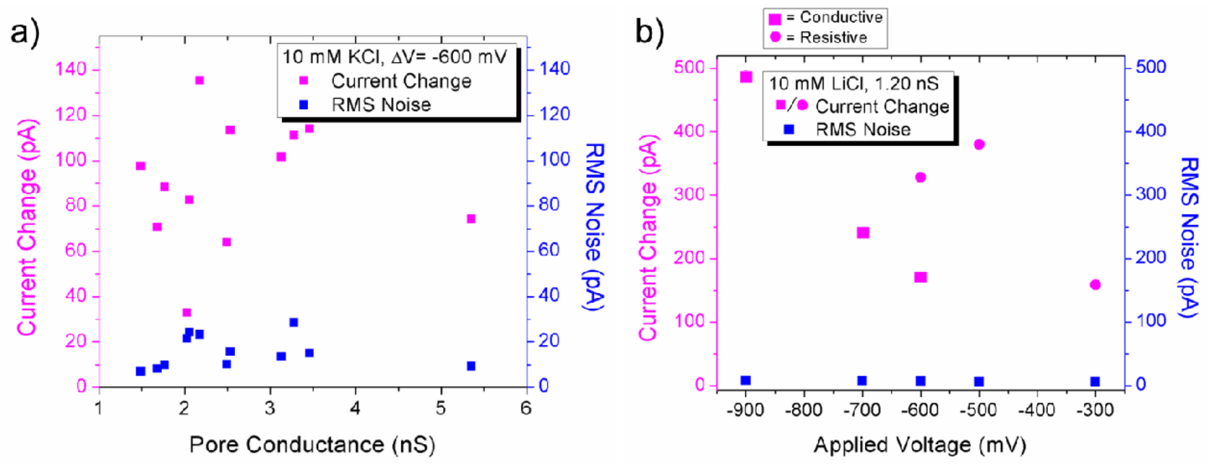

Supplementary Figure 5: **Relationship between current change, RMS noise, and pore conductance for 10 mM KCl + λ-DNA.** (**a**) At conductance values less than 2.0 nS, the RMS noise remains below 10 pA. We witness the current change increase as pore conductance increases from 2.0 nS. We propose that a larger influx of cations with larger pore sizes is the reason for this increase in current change. A possible outlier to this explanation resides at 5.4 nS. (**b**) Events stemming from 10 mM LiCl are further separated by CEs or REs. For all voltages, the RMS noise value was 7 ± 1 pA, suggesting that the main cause of difference in SNR is the change in current. We see a mostly linear relationship between the change in current and voltage applied with the exception of a polarity change occurring at -600 mV. Specifically, the CE amplitudes seen at -600 mV, -700 mV, and -900 mV linearly increase as the voltage increases in negativity. Likewise, we witness the same observation for REs occurring at -300 and -500 mV. The change in current with respect to voltage applied is mostly linear with the exception of a polarity change which occurs at -600 mV. Lastly, for this figure, we have only considered linearly translocating DNA. It is unlikely, but there is a small chance that DNA configurations other than linear were not excluded properly with the MATLAB analysis. Because DNA linearization has some dependence on voltage[6], it could possibly explain the non-linear relationship mostly occurring at -600 mV.

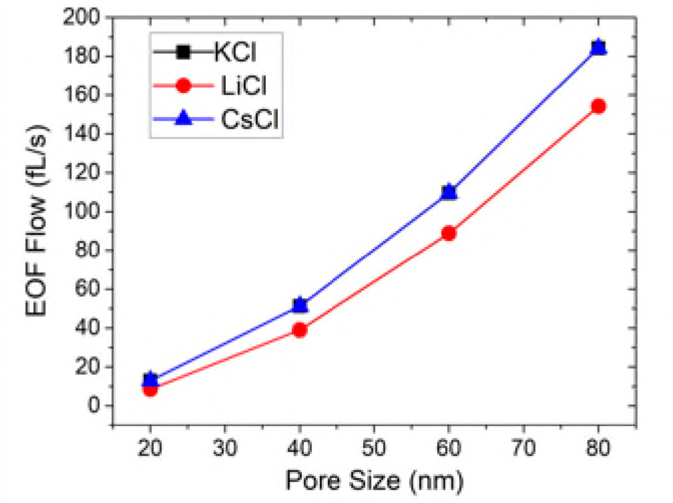

Supplementary Figure 6: COMSOL modelling demonstrating the rise in EOF-pumping with pore size.

Section 5: Finite Element Methods

Finite element modelling was developed using COMSOL Multiphysics. The nanopores geometries were built on the TEM images and pulling protocols achieved from the experimental studies. The pore diameters used in figures and text refer strictly to the internal diameters of the most constricting portion of the geometry. The corners of the pore were also curved with an arc radius of 4 nm to avoid anomalies typical of sharp corners. The meshing of the geometry was performed with boundary layers accentuated for increased resolution and accuracy of the ionic flux within these layers (1-10 nm from all surfaces). A conical nanopore with a 25 nm diameter pore and a 4° half-cone angle was used unless otherwise stated. The diffusion coefficients were considered 2E-9 [m^2/s] and 1.78E-9 [m^2/s] for the potassium (K^+) and chloride (Cl^-) ions, respectively. The Poisson, Nernst-Planck, and Navier-Stokes equations were simultaneously solved to model the ionic behavior in a 2D axisymmetric steady-state model. The Poisson's equation [$\nabla^2(V) = -\rho\nu/\varepsilon$] described the relationship between the electric potential and ion transport

mechanism. An important dimensional quantity in the Poisson equation is the Debye length, defined as $x_D = \sqrt{\frac{RT\varepsilon_o\varepsilon_s}{F^2 I}}$ where I is the ionic strength and F is Faraday's constant. Poissons equation can also be written as $\rho = F\sum_i c_i z_i$ where ρ is the space charge density of the fluidic domain. In COMSOL, the space charge density was specified within the *Electrostatics* module as well as the *Transport of Diluted Species* module as a volume force acting on the fluid (electroosmotic flow) and was defined specifically for binary electrolytes as: $\rho = N*e*z_1*c_1+N*e*z_2*c_2$ where for the electrolyte containing c_1 (K^+) and c_2 (Cl^-) ionic species, z and e were set as the valency and electron charge, respectively. The electrostatics boundary condition used for the glass was set at a surface charge density of -2E-2 [C/m^2] in the vicinity of the pore opening to consider the surface charge contributions. The electric potential was set as variable field and the initial values were defined as zero potential.

The Poisson-Nernst-Planck equation was solved for the transport properties and ionic fluxes using convection, diffusion, and migration terms. The equation was described as: $J_i = -D_i \nabla c_i - z_i \mu_{m,i} F c_i \nabla^2 V + c_i u$ where Ji, Di, c_i, u, $\mu_{m,i}$ and z_i are the ion flux, ion diffusion coefficient, concentration, fluid velocity, ion mobility and the charge number respectively. A no flux (J=0) condition was defined on the nanopore walls. The initial concentrations values of K^+ and Cl^- species were set to 10E-3 [mol/L] for the entire domain. The inlet and outlet of the fluidic chamber was defined using an open boundary condition and the concentrations of the bulk electrolyte. The open boundary condition allows for convective inflow and outflow to occur which is important since convection currents could affect the concentration of ions within the system. The concentration of any "inflow" is set at the bulk value of the concentration. The fluid flow and pressure were modeled by the Navier-Stokes's equation as: $\rho(u. \nabla)u = (-\nabla_p + \eta\, \nabla^2 u - F\,(\Sigma\, z_i\, c_i)\, \nabla\Phi$. The u and Φ are the position dependent velocity field and potential field, z_i and c_i are species i

charge and concentration in solution, ρ and η are the fluid density and dynamic viscosity, p is the pressure and F is the Faraday's constant. Initial values of zero were assigned to the velocity field and pressure. The boundary condition for the pore wall was set to be u=0 (no-slip). To model the fluid flow, the volumetric force on the fluid was defined as ions space charge density multiplied by the electric field vectors. The fluid velocity was averaged over a 2D line spanning the nanopore orifice width. Peak velocity was at the center of the pore due to the zero potential boundary condition for the pore walls. Velocity and pressure were specified as the boundary conditions for the inlet and outlet, respectively.

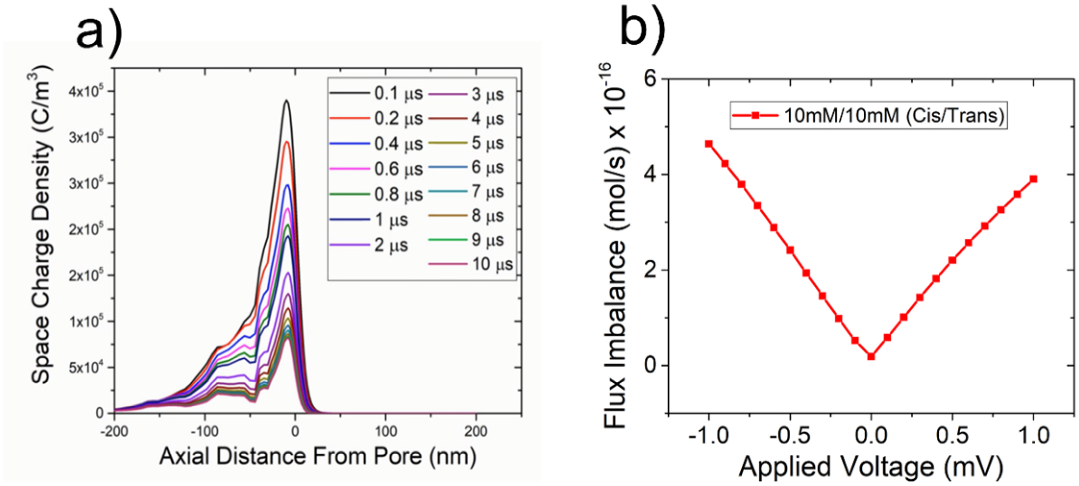

Supplementary Figure 7: (a) COMSOL modelling demonstrating the timescale of charge dispersion once EOF pumping is removed (voltage bias: -600 mV). The timescale of charging and discharging accumulated charge is fast (3-5 μs to reach steady state space charge density). (b) Flux imbalance under symmetric low salt conditions.

Section 6: Event Rate Calculation

The event rate was calculated using a technique previously described by *Wanunu et. al*[8]. Briefly, interevent time (δt) was calculated using a custom MATLAB code. Once interevent information was retrieved, a histogram was developed, and points corresponding to the histogram (δt and normalized counts) were plotted on a semilogarithmic graph. To calculate the event frequency in Supplementary Figure 2, a different approach was utilized to display more variables within the plot. Using this method, the interevent time for each data file (60 s) was calculated and summed together. The total number of events was then divided by the total interevent time to yield units of events per second for each depth.

It is important to note that each nanopipette was examined using an optical microscope prior to any experiments. This was performed to ensure the pore was free of any breaks, clogs, or air bubbles that may affect the quality of the current fluctuations. Our largest depth was recorded at 4.2 mm, meaning that the entire taper was submerged within the electrolyte solution. We speculate that the event frequency will not increase vastly above 40 Hz for depths exceeding the entire taper (i.e., the entire capillary), as that would entail the EOF capture zone to extend into the centimeter regime. Further validation of this model is needed but may provide a framework for future experiments.

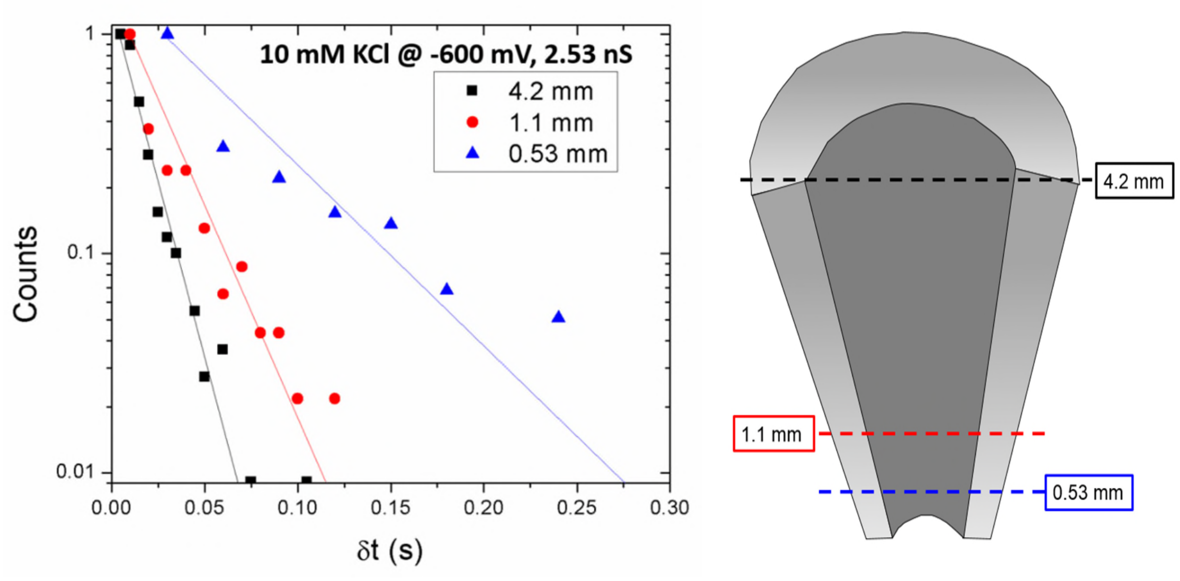

Supplementary Figure 8: Depth dependent study using 10 mM KCl + λ-DNA. Each condition describes how deep into the solution the nanopore resides. As the nanopore depth increases, a greater amount of the capture volume is exposed, and we witness an increase in event frequency. A descriptive illustration of the depth dependence study is on the right. The nanopore was mounted onto a manual linear stage actuator. The stage was raised and lowered by twisting the mechanical knob accordingly. Depth zero was identified by applying a small voltage into the pore and lowering the nanopore closer to the electrolyte bath. Once the current changed (in response to the circuit becoming connected), that location was denoted as the depth at 0 mm. From there, the mechanical knob was twisted to yield the following depths: 0.53, 1.1, and 4.2 mm (the entire taper).

Section 7: Event Extraction and Classification of Conformations

Events were extracted from raw current traces by subtracting the baseline variations, and using a thresholding algorithm, an event was flagged for further analysis. For each threshold crossing, the code would iteratively back-track and move forward until the baseline was reached. The full-width-half-max (FWHM) of the event was used for the event duration or dwell time. The maximum current (peak current) and area (in units of pA·s) for each event was also extracted.

These three event properties (event duration, peak amplitude, and area) were fed into a support vector machine classifier that was trained on user-defined folding states of the DNA. After running the classifier, all events were double checked to ensure classification was accurate.

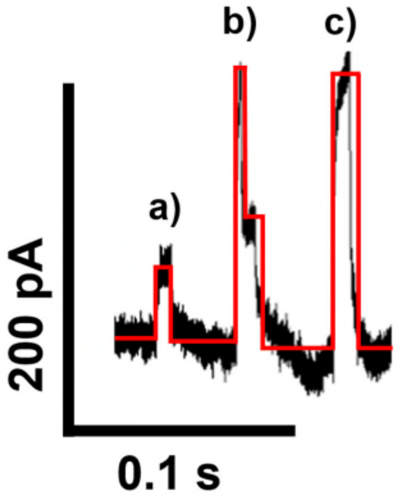

Supplementary Figure 9: Event Classification of DNA. A DNA molecule would translocate (a) linearly, (b) partially folded or (c) full-folded. Other conformations were negligible. Events concatenated together. Red lines are guides for the eye to recognize three different DNA configurations.

Section 8: Electroosmotic versus Electrophoretic Transport and Flux Imbalances

Flux imbalances differ from ion selectivity in that a flux imbalance can be produced through externally manipulating the pore size, salt concentration, voltage applied, or salt type (i.e., KCl, LiCl, CsCl) whereas ion selectivity stems from the properties of the pore itself. Under symmetric, low ionic strength conditions (10 mM KCl), EOF pumps cations into the pore, producing a flux imbalance in favor of K^+, yielding CEs when DNA translocates the pore. The surrounding pore environment can be altered to have a cationic flux imbalance when a concentration gradient is used (1 M KCl inside and 4 M KCl outside). While a negative voltage is

applied inside the pore, K^+ is pumped into the pore via EPF, producing CEs when DNA exits the pore. Similarly, the pore environment can have a flux imbalance in favor of anions, where 4 M KCl is inside the pore and 1 M KCl is outside. When a positive voltage is applied to attract DNA, Cl^- is pumped into the pore. Upon translocation, REs are generated. Thus, we conclude that a flux imbalance in favor of cations produces CEs and a flux imbalance in favor of anions produces REs.

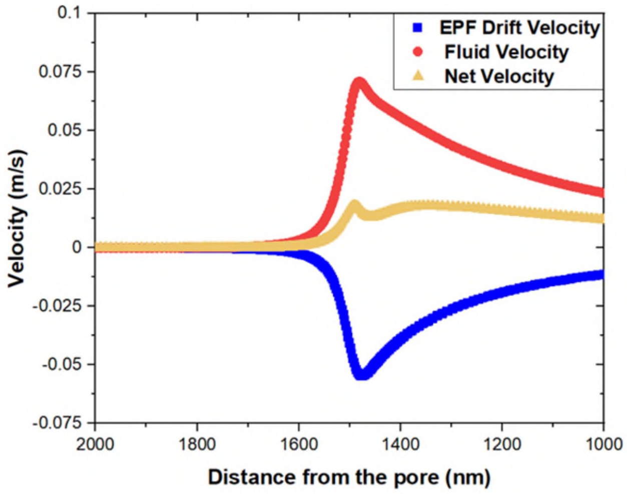

Supplementary Figure 10: COMSOL simulations reveal that EOF velocity is greater than EPF drift velocity. Therefore, the net velocity is towards the pore, allowing DNA to translocate via EOF. The electrolyte parameters were based on 10 mM KCl. The pore was modelled as having a half-cone (taper) angle of 8 degrees which was not critical to observing EOF dominance. EOF dominance was also found across multiple pore sizes spanning the range used experimentally (70 nm diameter was used for this figure). The pore surface parameters were based on quartz. Lithium chloride translocations were also observed in borosilicate nanopipettes which have a lower surface charge but were not modelled here. The electrophoretic mobility used for λ-DNA was 3.0×10^{-4} $cm^2\ V^{-1}s^{-1}$. The results were also not dependent on applied voltage as variations in voltage scale both velocities equally (V= -700 mV is shown here).

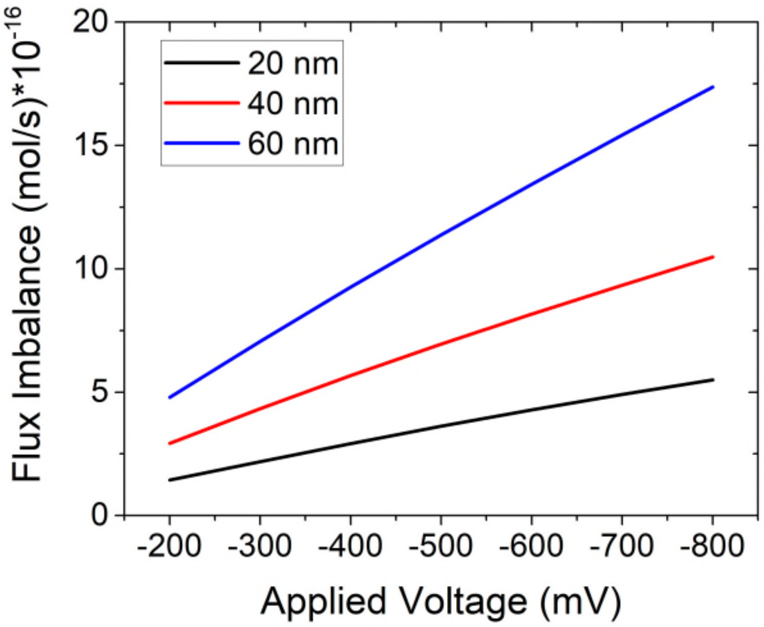

Supplementary Figure 11: COMSOL simulations reveal flux imbalances are voltage and pore size dependent. Flux imbalance was found by integrating the difference in cation flux and anion flux across the pore lumen. Positive flux imbalance indicates that the cation is dominant. The conditions for the simulation included a pore wall with surface charge of 20 mC/m^2 and a salt concentration of 10 mM. The salt condition used for this figure was KCl.

Section 9: Streaming Current

Although the ionic diffusion coefficients and electrophoretic mobilities encapsulate basic transport properties, all the while being utilized as variables in the finite element simulations, they neglect the geometric size of the ions and therefore the packing density/strength on oppositely charged surfaces. To understand the link between electro-hydrodynamics and Debye layer screening of the quartz surface charge, streaming current measurements were used as a proxy for cation mobility within the diffuse ion layer. Contrary to EOF, where mobile ions drag fluid, streaming currents measure the fluid's ability to drag along ions co-axial to the fluid motion[9]. A pressure bias was used to generate a streaming current and the resulting data can be seen in

Supplementary Figure 10. Negative pressures generate a flow into the nanopore and in the same direction as EOF in our experiments. We see that larger pressures create larger streaming currents. Interestingly, LiCl has significantly higher streaming currents compared KCl and CsCl at negatively biased pressures. The same pore (1.3 nS in 10 mM KCl) was used for all measurements to reduce variability due to different pore sizes.

When an electrolyte solution is traveling through the negatively charged glass nanopore via pressure, an electrical double layer is constructed. In this layer, there is an increase in cation (K^+, Li^+, or Cs^+) concentration and a decrease in chloride concentration locally at the pore surface. The resulting electrical double layer produced by streaming currents is comparable to the Debye screening layer and the streaming potential is directly related to the zeta potential[10]. Thus, by performing streaming current measurements, we obtain information relating to the Debye length and zeta potential at low ionic strength conditions.

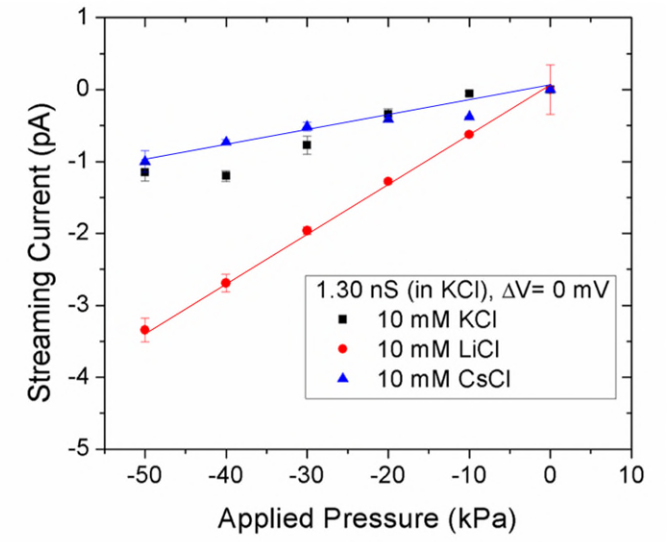

Supplementary Figure 12: Experimental data showing the streaming potential variance among 10 mM KCl, LiCl, and CsCl. Error bars made from taking the average current of three segments of data at one pressure point.

Section 10: Translocation Characteristics in LiCl

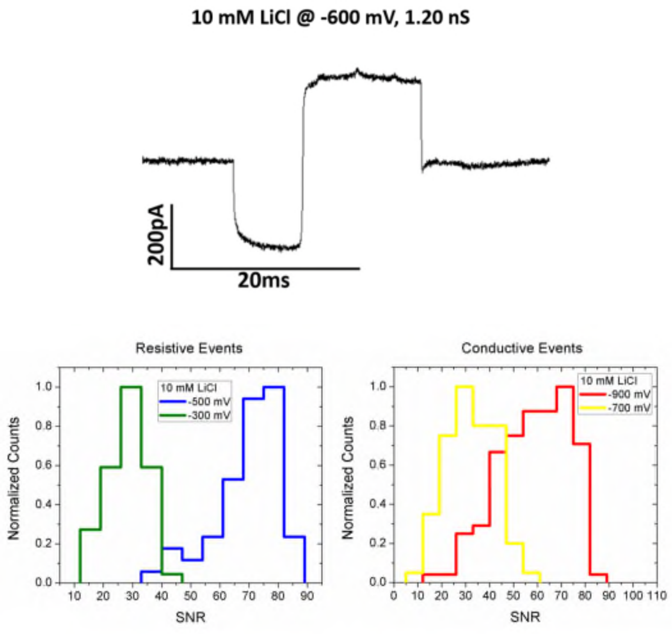

Supplementary Figure 13: **A typical current trace of events occuring at -600 mV.** Interestingly, we see a biphasic, nearly eqiphasic, event t -600 mV under low salt conditions (top figure). We propose that before translocation, DNA entering the flow field might perturb the current, thus affecting the hydrodynamic flow and pore occupancy. Resistive (bottom left) and conductive (bottom right) event voltage dependence on SNR in 10 mM LiCl.

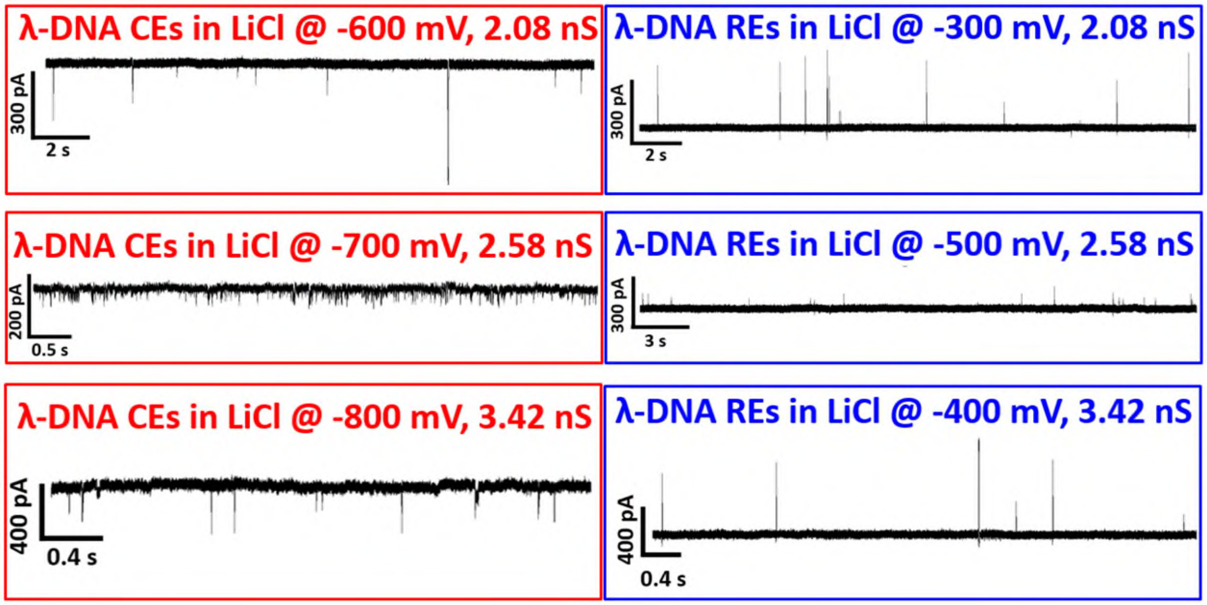

Supplementary Figure 14: Current traces of λ-DNA translocating in three different pores in 10 mM LiCl. DNA was observed translocating through three nanopores at various applied voltages. For each pore, REs can be seen at lower negative voltages. Oppositely, CEs are witnessed at larger negative voltages. On the left (red) are three current traces containing CEs and, on the right (blue), REs are shown. Current traces from the same row are taken from the same pore.

Section 11: Measurements in CsCl

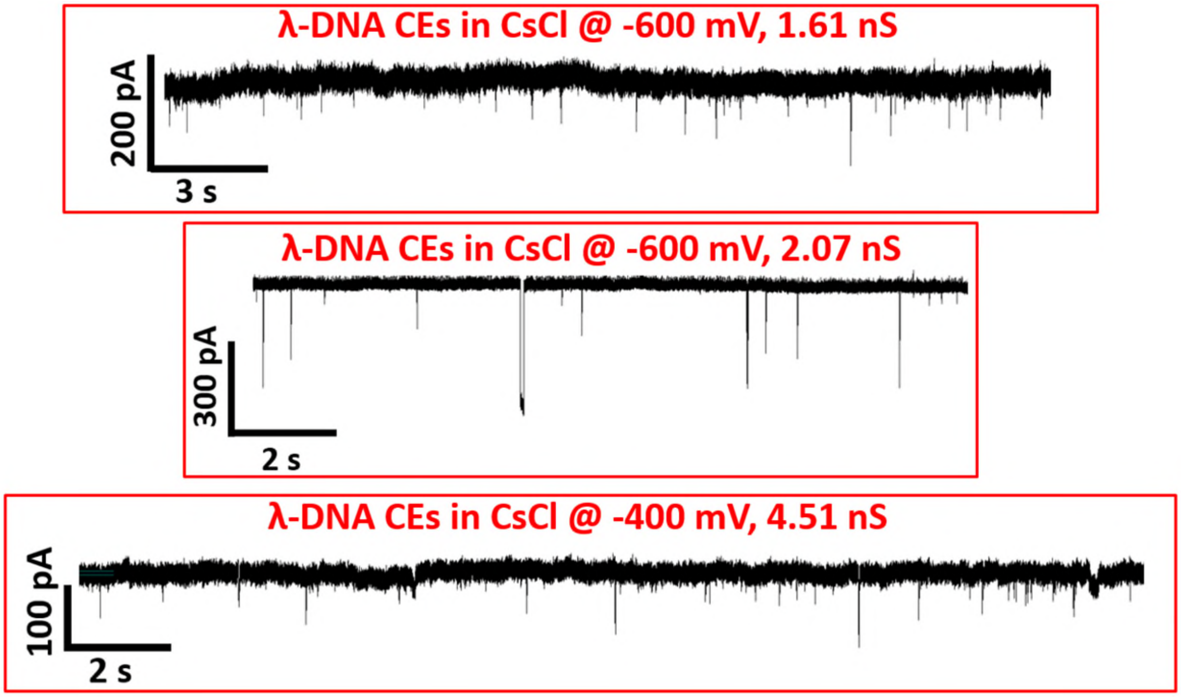

Supplementary Figure 15: Current traces of λ-DNA translocating in three different pores in 10 mM CsCl. Similar to KCl, we do not see either a voltage dependence or pore size dependence on event shape. Differently sizes pores were fabricated and all yielded CEs. This, along with Supplementary Figure 3e, indicates neither a pore size or voltage dependence for witnessing CEs/REs in 10 mM CsCl.

Section 12: Time-Dependent Electric field and Flux Models

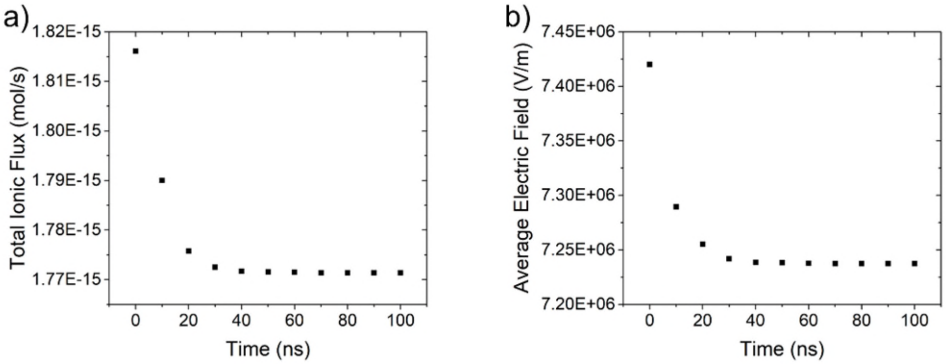

Supplementary Figure 16: Time-dependent flux and electric field magnitude inside a nanopore. The simulation results are for a 16nm pore (internal diameter), 10mM KCl, surface charge of 10 mC/m^2, and a voltage bias of -600mV (applied internal to the nanopipette).

References

1. Reiner, J. E., Kasianowicz, J. J., Nablo, B. J. & Robertson, J. W. Theory for polymer analysis using nanopore-based single-molecule mass spectrometry. *Proceedings of the National Academy of Sciences* **107**, 12080–12085 (2010).
2. Zhang, Y. *et al.* Ionic current modulation from DNA translocation through nanopores under high ionic strength and concentration gradients. *Nanoscale* **9**, 930–939 (2017).
3. Smeets, R. M. M. *et al.* Salt Dependence of Ion Transport and DNA Translocation through Solid-State Nanopores. *Nano Lett.* **6**, 89–95 (2006).
4. Steinbock, L. J., Otto, O., Chimerel, C., Gornall, J. & Keyser, U. F. Detecting DNA Folding with Nanocapillaries. *Nano Lett.* **10**, 2493–2497 (2010).
5. Plesa, C. *et al.* Direct observation of DNA knots using a solid-state nanopore. *Nature Nanotech* **11**, 1093–1097 (2016).
6. Chen, P. *et al.* Probing Single DNA Molecule Transport Using Fabricated Nanopores. *Nano Lett.* **4**, 2293–2298 (2004).
7. Plesa, C., van Loo, N., Ketterer, P., Dietz, H. & Dekker, C. Velocity of DNA during Translocation through a Solid-State Nanopore. *Nano Lett.* **15**, 732–737 (2015).
8. Wanunu, M., Morrison, W., Rabin, Y., Grosberg, A. Y. & Meller, A. Electrostatic focusing of unlabelled DNA into nanoscale pores using a salt gradient. *Nature Nanotech* **5**, 160–165 (2010).
9. *Colloid and Interface Chemistry for Water Quality Control*. (Elsevier, 2016). doi:10.1016/C2014-0-02506-3.

10. West, A. Chapter 3 - Experimental Methods to Investigate Self-Assembly at Interfaces. in *Interface Science and Technology* (ed. Ball, V.) vol. 21 131–241 (Elsevier, 2018).
